# *Anopheles* bionomics, insecticide resistance and malaria transmission in southwest Burkina Faso: A pre-intervention study

**DOI:** 10.1371/journal.pone.0236920

**Published:** 2020-08-03

**Authors:** Dieudonné Diloma Soma, Barnabas Mahugnon Zogo, Anthony Somé, Bertin N’Cho Tchiekoi, Domonbabele François de Sales Hien, Hermann Sié Pooda, Sanata Coulibaly, Jacques Edounou Gnambani, Ali Ouari, Karine Mouline, Amal Dahounto, Georges Anicet Ouédraogo, Florence Fournet, Alphonsine Amanan Koffi, Cédric Pennetier, Nicolas Moiroux, Roch Kounbobr Dabiré

**Affiliations:** 1 Institut de Recherche en Sciences de la Santé (IRSS), Bobo-Dioulasso, Burkina Faso; 2 Université Nazi Boni, Bobo-Dioulasso, Burkina Faso; 3 MIVEGEC, Université de Montpellier, CNRS, IRD, Montpellier, France; 4 Institut Pierre Richet (IPR), Bouaké, Côte d’Ivoire; 5 Université d’Abomey Calavi, Abomey-Calavi, Benin; 6 Université de Dédougou, Dédougou, Burkina Faso; Swiss Tropical and Public Health Institute, SWITZERLAND

## Abstract

**Background:**

Twenty-seven villages were selected in southwest Burkina Faso to implement new vector control strategies in addition to long lasting insecticidal nets (LLINs) through a Randomized Controlled Trial (RCT). We conducted entomological surveys in the villages during the dry cold season (January 2017), dry hot season (March 2017) and rainy season (June 2017) to describe malaria vectors bionomics, insecticide resistance and transmission prior to this trial.

**Methods:**

We carried out hourly catches (from 17:00 to 09:00) inside and outside 4 houses in each village using the Human Landing Catch technique. Mosquitoes were identified using morphological taxonomic keys. Specimens belonging to the *Anopheles gambiae* complex and *Anopheles funestus group* were identified using molecular techniques as well as detection of *Plasmodium falciparum* infection and insecticide resistance target-site mutations.

**Results:**

Eight *Anopheles* species were detected in the area. *Anopheles funestus s*.*s* was the main vector during the dry cold season. It was replaced by *Anopheles coluzzii* during the dry hot season whereas *An*. *coluzzii* and *An*. *gambiae s*.*s*. were the dominant species during the rainy season. Species composition of the *Anopheles* population varied significantly among seasons. All insecticide resistance mechanisms (*kdr-w*, *kdr-e* and *ace-1* target site mutations) investigated were found in each members of the *An*. *gambiae* complex but at different frequencies. We observed early and late biting phenotypes in the main malaria vector species. Entomological inoculation rates were 2.61, 2.67 and 11.25 infected bites per human per month during dry cold season, dry hot season and rainy season, respectively.

**Conclusion:**

The entomological indicators of malaria transmission were high despite the universal coverage with LLINs. We detected early and late biting phenotypes in the main malaria vector species as well as physiological insecticide resistance mechanisms. These data will be used to evaluate the impact of complementary tools to LLINs in an upcoming RCT.

## Introduction

The World Health Organization (WHO) has reported 228 million malaria cases and 405 000 deaths worldwide in 2018 [1]. Significant progress has been made in the era of malaria control and elimination between 2000 and 2017 in all regions of the world [2]. The number of malaria cases worldwide has decreased by 62% between 2000 and 2015 but seems to rebound since 2016 [2–4]. Sub-Saharan Africa accounted for about 93% of cases and deaths in 2017 [1]. In 2017, the health ministry of Burkina Faso recorded 11.9 million cases and 4144 deaths attributed to malaria [5].

Malaria control is mainly based on symptomatic and preventive treatments (with artemisinin-based combination therapies: ACTs) and vector control. Vector control aims at reducing malaria transmission by targeting *Anopheles* mosquitoes that transmit *Plasmodium* parasites. Core vector control measures rely mostly on the mass distribution of long-lasting insecticidal nets (LLINs) and high coverage of indoor residual spraying (IRS) to reduce the risk of malaria infection by targeting indoor biting mosquitoes [4]. It was estimated that LLINs have contributed to 68% of the decline in malaria cases observed between 2000 and 2015 in Africa [6] despite moderate use rates (LLIN use rate reached 55% in 2015 in sub-Saharan Africa [7]. However, the emergence of physiological [8–10] and behavioral [11,12] insecticide resistance mechanisms in *Anopheles* mosquitoes, as observed in most parts of Africa, could both compromise the effectiveness of LLINs and explain the recent rebound in malaria cases. Consequently, there is an urgent need for increasing or complementing the protection provided by the LLINs. Complementary strategies exists but before being included into strategic plans by national malaria control programs (NMCPs), supported by international donors and implemented in endemic countries, they need to be evaluated through rigorous and independent process [13]. In this context, Institut de Recherche en Sciences de la Santé (IRSS), Institut de Recherche pour le Développement (IRD) and Institut Pierre Richet (IPR) have been funded to conduct the REACT (Gestion de la **RE**sist**A**nce aux inse**CT**icides au Burkina Faso et en Côte d’Ivoire) project to evaluate in Burkina Faso and Côte d’Ivoire four complementary strategies to LLINs trough a randomized controlled trial (RCT). These strategies included: i) larviciding with *Bacillus thuringiensis israelensis* (Bti) to target immature stages of *Anopheles* species, ii) Indoor residual spraying (IRS) with pirimiphos-methy to target endophilic malaria vectors, iii) information, education, community (IEC) to improve LLINs use iv) ivermectin administration to animals, a One health approach to tackle zoophagic behavior of malaria vectors and to improve animal health. The current study conducted entomological surveys to describe malaria vector bionomics, insecticide resistance and transmission prior to the implementation of the REACT RCT.

## Materials and methods

### Study site and design

The study was conducted in the Diébougou health district located in southwest Burkina Faso. The natural vegetation is mainly wooded savannah dotted with clear forest gallery. The climate is tropical with two seasons: one dry season from October to April and one rainy season from May to September. The average annual rainfall is about 1200 mm. The dry season is “cold” from December to February (with average minimal and maximal temperatures of 18°C and 35°C, respectively) and “hot” from March to April (with average minimal and maximal temperatures of 25°C and 40°C, respectively). Agriculture (cotton growing and cereals) is the main economic activity in the area, followed by artisanal gold mining and production of coal and wood [14, 15]. The study took place in 27 villages that were selected considering accessibility during the rainy season, a size of 200–500 inhabitants per village and a distance between two villages higher than two kilometers. According to a census carried out by our team in August 2016 one month after a universal distribution by the NMCP, the proportion of households with at least one LLIN and with at least one LLIN for every two inhabitants were 96.15% and 71.17%, respectively with 8680 inhabitants in the 27 selected villages. The proportion of households with access to an LLIN in their household was 87.60%. We calculated these indicators according to WHO formulas [16].

### Mosquito collection and determination

We carried out three rounds of mosquito collection in January 2017 (dry cold season), March 2017 (dry hot season) and June 2017 (rainy season) using Human Landing Collections (HLC). The procedure for conducting HLC was for a person to sit on a stool, and mosquitoes to alight on his exposed legs where they were then collected using a hemolysis tube [17, 18]. Mosquitoes were collected from 17:00 to 09:00 both indoors and outdoors at 4 sites per village (sites of collection remained the same during the three surveys). We performed collections in three villages simultaneously for one night leading to 9 nights of collections needed to survey all 27 villages during each survey. Due to a long duration of collection (16h), two teams of 8 collectors worked in each village. The first team worked from 17:00 to 01:00 when they were replaced by the second team from 01:00 to 09:00. Collectors were rotated among the collection points every hour. Indoor collection points were rooms that meet the following criteria: being usually inhabited; quiet without excessive movement of peoples; open to the outside through a door or a window. Outdoor collection was conducted in areas usually occupied by people but are sheltered from wind, traffic, fires and are not large meeting areas. The distance between collection sites was at least 50 m. The distance between indoors and outdoors collection points in one site was at least 10 m to minimize competition between mosquito collectors. Mosquitoes were collected in individual tubes plugged with cotton and stored in hourly bags. Independent staff supervised rotation of the mosquito collection and regularly checked for the quality of the mosquito collection. The following criteria were checked: respected collection location, collector at his post, collector awake, collector in a correct position, collector adequately dressed, correct hourly bags used. If one of the criteria was not respected, required arrangements were immediately made by the supervisor.

### Morphological identification and dissection

Mosquitoes were morphologically identified where possible in the field to genus and species levels using morphological keys [19, 20]. A subsample of 100 non blood-fed *Anopheles spp*. individuals was randomly selected per survey and per village and dissected to identify their parity state (parous or nulliparous) [21]. Parous female are those that have laid eggs at least once. All females belonging to the *Anopheles* genus were stored in individual tubes with silicagel and preserved at -20°C for further analyses.

### Molecular analysis

DNA extracted from head-thorax of *Anopheles spp*. individuals was used to detect *Plasmodium falciparum* infection using quantitative polymerase chain reaction (PCR) assay [22]. Individuals belonging to the *Anopheles gambiae* complex and the *Anopheles funestus group* were identified to species by PCR [23–25].

PCR assay were carried out on all mosquitoes belonging to the *An*. *gambiae* complex to detect the L1014F (*kdr-w*) [26], the L1014S (*kdr-e*) [27] and the G119S (*ace-1*) [28] mutations. *kdr-w* and *kdr-e* confer insecticide resistance to pyrethroids whereas *ace-1* confers resistance to carbamates and organophosphates.

### Parameters measured

We calculated the human biting rate (HBR; the number of vectors’ bites per human per night (b.h^-1^.n^-1^), the sporozoite infection rate (SIR: the proportion of *Anopheles* infected by *P*. *falciparum*), the entomological inoculation rate (EIR; the number of infected bites per human per night (ib.h^-1^.n^-1^), the endophagy rate (ER; the proportion of *Anopheles* females collected indoors) and the parous rate (PR; the proportion of parous females over the total dissected) for each *Anopheles* species and for overall *Anopheles spp*. From PRs, we deduced daily survival rates (*p*) using the Davidson’s method [29] ($p=\sqrt[{g}]{PR}$ with the duration of the gonotrophic cycle *g* assumed to be 2 days). Knowing survival rates, we were able to estimate the proportion *P*_*d*_ of the vector population that reach the epidemiologically dangerous age according to the formulae given by MacDonald (Expression 5 p.16 in [30]: *P*_*d*_ = *p*^*n*^) assuming the sporogony duration *n* of malaria parasites in mosquitoes takes 11 days after which the parasites can be transmitted back to humans.

### Statistical analyses

We assessed HBR, ER, SIR, EIR and PR using generalized mixed effect models (GLMM) with collection points and villages as nested random intercept. All models were fitted using the ‘glmer’ function of the package ‘lme4’ [31] using R software [32]. For all models, we performed backward stepwise deletion of the fixed terms followed by Likelihood ratio tests. Term removals that significantly reduced explanatory power (*p* < 0.05) were retained in the minimal adequate model. We used the post-hoc Tukey method to do multiple comparison among modalities of the fixed terms and calculated estimated marginal means (EMM) using the ‘emmeans’ function of the ‘emmeans’ package [33]. We considered main effects and interactions of the fixed terms in all the models.

We performed HBR and EIR analyses using negative-binomial models fitted on nightly counts of all *Anopheles* and of *Anopheles* individuals found to be infected by *P*. *falciparum*, respectively. Fixed terms were the season (dry cold, dry hot or rainy) and the collection position (indoor or outdoor). The results of final models are presented (S1 File).

We performed SIR, ER and PR analyses using binomial models fitted on individual status of the *Anopheles* (infected *vs*. uninfected, collected indoors *vs*. outdoors or parous *vs*. nulliparous, respectively). Fixed terms for these binomial models were the season and the individual species. Rate ratio (RR) for Negative Binomial models and Odds ratio (OR) for Binomial models were computed with 95% confidence intervals.

The relationship between *Anopheles* species composition and seasons was tested using a Fisher’s exact test.

We assessed nightly activity of each major *Anopheles* species by comparing their Median Catching Time (MCT), which represents the time at which 50% of the individuals were collected [11], using a Kruskal-Wallis test and a Dunn’s post-hoc test for multiple comparisons (function ‘dunnTest’ of the ‘VIA’ package) in R [34].

We compared the genoptypes frequencies of *kdr* and *ace-1* mutations among seasons and species using the G-test [35] implemented in Genepop 4.7 and run in R [36]. Genotypic frequencies at the *kdr* and *ace-1* mutations were tested for conformity to Hardy-Weinberg equilibrium using the “Exact HW test” [37]. In case of disequilibrium, we tested heterozygote excess and deficiency using the score test [38]. We tested the relationship between infectiousness and the genotypes for the mutations *kdr-w*, *kdr-e* and *ace1* using Pearson’s Chi-square tests (or a Fisher’s exact test when one or more count were < 5).

### Ethics approval and consent to participate

The protocol of this study was reviewed and approved by the Institutional Ethics Committee of the Institut de Recherche en Sciences de la Santé (IEC-IRSS) and registered as N°A06/2016/CEIRES. Mosquito collectors and supervisors gave their written informed consent. They received a vaccine against yellow fever as a prophylactic measure. Collectors were treated free of charge for malaria according to WHO recommendations.

## Results

### *Anopheles* densities and composition

**Fig 1:**
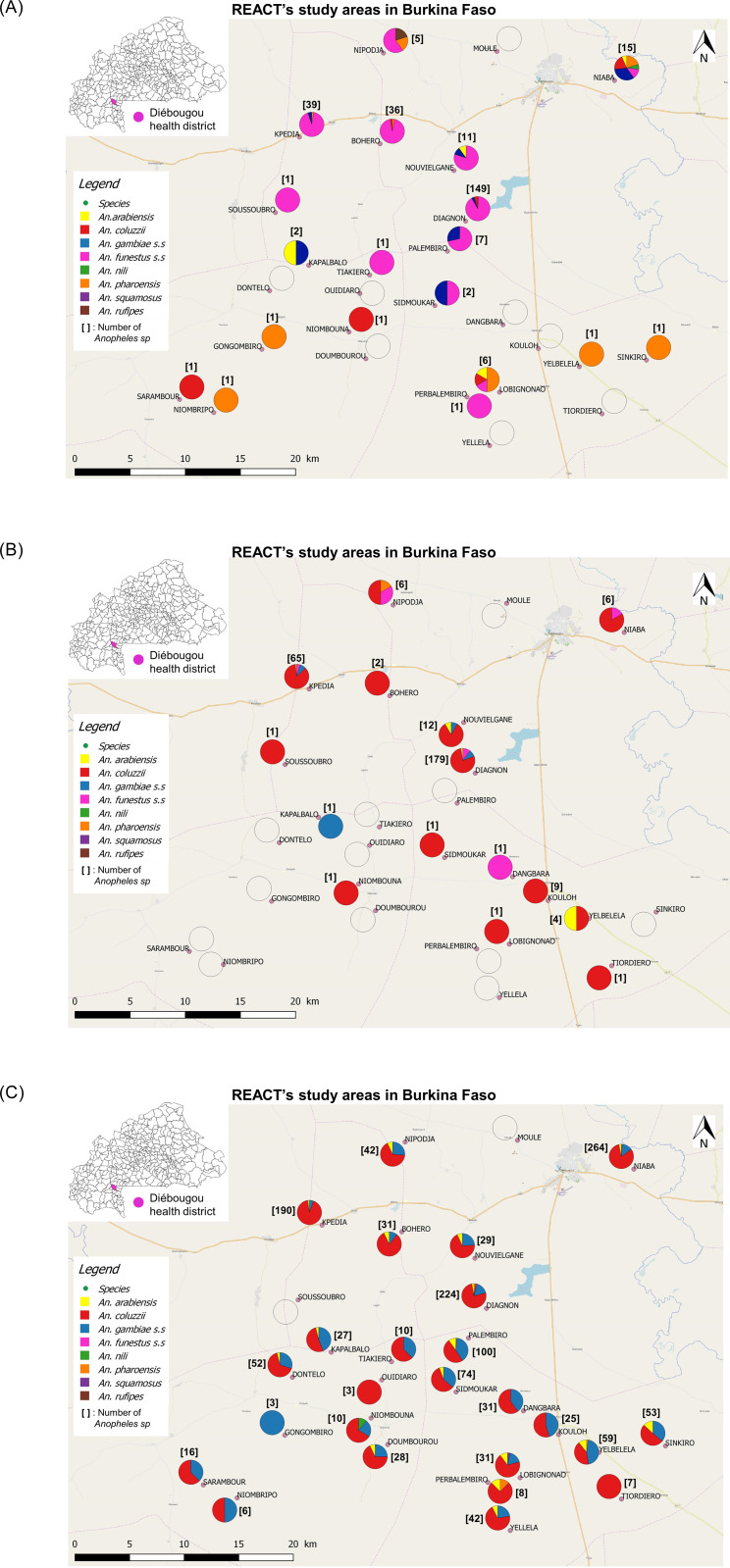
**A**. Map of *Anopheles* densities and composition during dry cold season. Top-left box shows the location of the Diébougou health district in Burkina Faso. Background was obtained freely from openstreetmap.org. **B**. Map of *Anopheles* densities and composition during dry hot season. Top-left box shows the location of the Diébougou health district in Burkina Faso. Background was obtained freely from openstreetmap.org. **C**. Map of *Anopheles* densities and composition during rainy season. Top-left box shows the location of the Diébougou health district in Burkina Faso. Background was obtained freely from openstreetmap.org.

**Fig 2:**
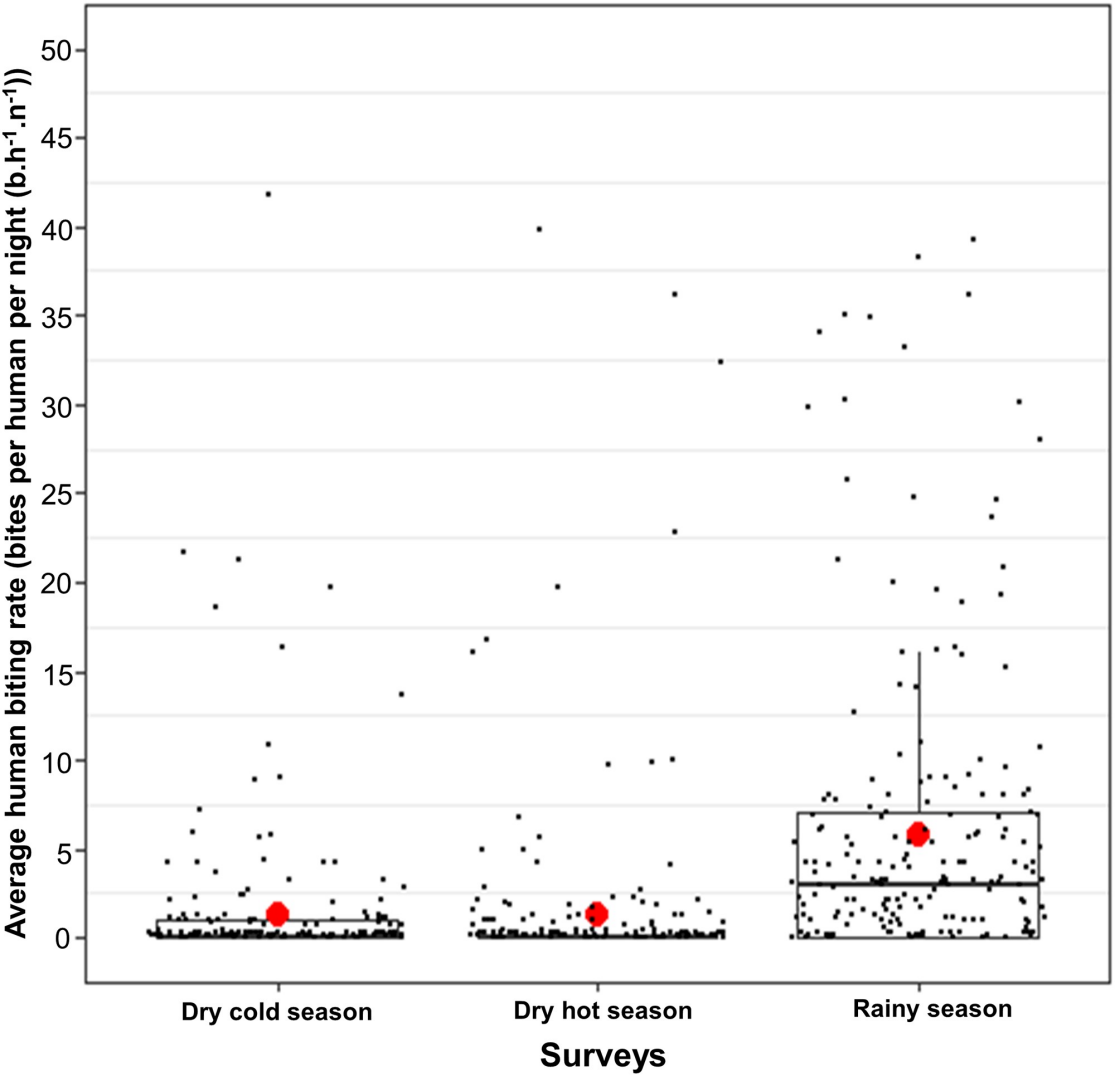
Human biting rates of *Anopheles* mosquitoes measured during dry cold, dry hot and rainy seasons in 27 villages of the Diébougou health district, Burkina Faso. Boxes indicate inter-quartile range (IQR) and median of human biting rates recorded at each season. Whiskers indicate 2.5–97.5 percentiles. The upper whisker extends from the hinge to the largest value no further than 1.5 * IQR from the hinge. Black small dots indicate HBR recorded at each collection point and red dots show the mean HBR per season.

During the three surveys, we collected a total of 2591 mosquitoes belonging to four genus: *Anopheles spp*. (n = 1936, 74.72%), *Aedes spp*. (n = 481, 18.56%), *Culex spp*. (n = 161, 6.21%) and *Mansonia spp*. (n = 13, 0.50%) (Table 1). We successfully identified by PCR 92.84% (1530/1648) of the *An*. *gambiae s*.*l*. individuals and 96.15% (250/260) of the members of the *An*. *funestus group*. Among the 1530 *Anopheles* individuals morphologically identified as members of the *An*. *gambiae* complex and proceeded by PCR, 1131 were *An*. *coluzzii*, 325 were *An*. *gambiae s*.*s*. and 74 were *An*. *arabiensis*. *Anopheles* morphologically identified as members of the *An*. *funestus group* and successfully proceeded by PCR were all (n = 250) *An*. *funestus s*.*s*. Other *Anopheles* species found during our surveys were *Anopheles nili* (n = 3), *Anopheles pharoensis* (n = 23), *Anopheles rufipes* (n = 1) and *Anopheles squamosus* (n = 1) (Fig 1A–1C). The average HBR of *Anopheles spp*. mosquitoes was 1.30 and 1.34 b.h^-1^.n^-1^ (bites per human per night) during dry cold and dry hot season (RR [95% CI] = 1.15 [0.78, 1.71], Tukey’s p = 0.67), respectively and these were significantly lower than 6.31 b.h^-1^.n^-1^ during the rainy season (RR = 0.090 [0.063, 0.130], Tukey’s p< 0.0001 and RR = 0.078 [0.054, 0.114], Tukey’s p< 0.0001, respectively for dry cold and dry hot season, when compared to rainy season) (Fig 2).

**Table 1:** Abundance and diversity of mosquito species in the Diébougou area during pre-intervention surveys.

	Dry cold season		Dry hot season		Rainy season		Total
Mosquito species	abundance (216 human-nights)	% of human-night with > = 1 ind.	abundance (216 human-nights)	% of human-night with > = 1 ind.	abundance (216 human-nights)	% of human-night with > = 1 ind.	
*Aedes aegypti*	2	0.93	1	0.46	103	20.83	106
*Aedes africanus*	0	0.00	0	0.00	6	1.85	6
*Aedes fowleri*	0	0.00	0	0.00	100	22.22	100
*Aedes furcifer*	0	0.00	0	0.00	101	18.52	101
*Aedes lutheocephalus*	0	0.00	0	0.00	16	6.94	16
*Aedes opok*	0	0.00	0	0.00	16	4.17	16
*Aedes vexans*	0	0.00	0	0.00	89	16.67	89
*Aedes vittatus*	0	0.00	6	2.78	41	12.04	47
*Anopheles funestus*	230	17.13	25	5.56	5	1.85	260
*Anopheles gambiae s*.*l*.	35	11.57	264	20.37	1349	74.07	1648
*Anopheles nili*	1	0.46	0	0.00	2	0.93	3
*Anopheles pharoensis*	14	6.02	1	0.46	8	3.24	23
*Anopheles rufipes*	1	0.46	0	0.00	0	0.00	1
*Anopheles squamosus*	0	0.00	0	0.00	1	0.46	1
*Culex cinereus*	1	0.46	9	1.85	15	2.78	25
*Culex decens*	0	0.00	1	0.46	2	0.46	3
*Culex poicilipes*	0	0.00	1	0.46	1	0.46	2
*Culex quinquefasciatus*	21	5.09	42	9.26	62	8.33	125
*Culex univittatus*	1	0.46	0	0.00	5	1.39	6
*Mansonia africana*	4	0.93	4	1.85	0	0.00	8
*Mansonia uniformis*	2	0.46	0	0.00	3	1.39	5
Total	312		354		1925		2591

Ind: individuals.

During the dry cold season, *An*. *funestus s*.*s* was the most abundant species representing 78.64% (n = 221/281), followed by *An*. *gambiae s*.*s* (6.04%, n = 17/281), *An*. *coluzzii* (4.98%, n = 14/281) and *An*. *pharoensis* (4.98%, n = 14/281) (Fig 1A). The other species were found at very low percentages (~1%) (Fig 1A). The relative abundance and species composition of the *Anopheles* population varied from one village to another. We collected *Anopheles* mosquitoes in 19 villages out of the 27 surveyed with the highest densities registered in Diagnon (n = 149, 4 species) and Kpédia (n = 39, 3 species) (Fig 1A). We observed the highest *Anopheles* species diversity in Niaba (n = 15, 6 species) where six species were identified. At the opposite in nine villages, only one species of *Anopheles* was found (either *An*. *funestus s*.*s*, *An*. *coluzzii* or *An*. *pharoensis*).

During the dry hot season, *An*. *coluzzii* (75.86%, n = 220/290) almost totally replaced *An*. *funestus s*.*s* (8.27%, n = 24/290) while proportions of other species do not vary substantially. During this season, *Anopheles* mosquitoes were collected in only 15 villages (Fig 1B). Highest densities and species diversities were observed in Diagnon (n = 179, four species) and Kpédia (n = 65, four species) (Fig 1B). We identified three *Anopheles* species in both Nouvielgane and Nipodja villages and two *Anopheles* species in Yelbelela and Niaba. In all other villages, only one *Anopheles* species was present at low density.

During the rainy season, *An*. *coluzzii* remained the major species (65.71%, n = 897/1365) followed by *An*. *gambiae s*.*s* (20.95%, n = 286/1365) and *An*. *arabiensis* (4.54%, n = 62/1365). During this season, *An*. *funestus s*.*s* fall under 1% of the total. We collected *Anopheles spp*. mosquitoes in 25 villages out of the 27 surveyed (Fig 1C). The highest densities were observed in Niaba (n = 264, four species), Diagnon (n = 224, six species) and Kpédia (n = 190, three species). We identified four *Anopheles* species in Lobignonao (n = 31), Palembiro (n = 100) and Yelbelela (n = 59), two *Anopheles* species in Dangbara (n = 31), Kouloh (n = 25), Niombripo (n = 6), Sarambour (n = 16) and Tiakiero (n = 10), and one *Anopheles* species in Gongombiro (n = 3), Ouidiaro (n = 3) and Tiordiero (n = 7). In the remaining villages, three *Anopheles* species were identified. No *Anopheles spp* mosquitoes were collected in Sousoubro and Moulé (Fig 1C).

Species composition of the *Anopheles* population varied significantly among seasons (Fisher’s exact test p = 0.0005).

### Mosquito biting behavior

Overall, endophagy rate (ER) of *Anopheles spp*. [95% CI] was 63.23% [57.50–68.96], 50.18% [44.27–56.09] and 57.18% [54.44–59.90] during the dry cold, dry hot and rainy seasons, respectively (Table 2).

**Table 2:** *Anopheles* species composition and abundance.

Species	Dry cold season			Dry hot season			Rainy season		
	Indoor^1^	Outdoor^1^	Total^2^	Indoor^1^	Outdoor^1^	Total^2^	Indoor^1^	Outdoor^1^	Total^2^
*An*. *arabiensis*	2	2	4	2	6	8	31	31	62
*An*. *coluzzii*	5	9	14	106	114	220	525	372	897
*An*. *gambiae s*.*s*	14	3	17	16	6	22	159	127	286
*An*. *funestus s*.*s*	146	75	221	14	10	24	3	2	5
*An*. *nili*	1	-	1	-	-		1	1	2
*An*. *pharoensis*	3	11	14	-	1	1	2	6	8
*An*. *rufipes*	1	-	1	-	-		-	-	-
*An*. *squamosus*	-	-		-	-		-	1	1
Total	172	100	272	138	137	275	721	540	1261
% Indoors [95% CI]	63.23 [57.50–68.96]			50.18 [44.27–56.09]			57.17 [54.44–59.90]		

^1^ 108 human-nights of collection;

^2^ 216 human-nights of collection.

During dry cold season, *An*. *funestus s*.*s* (EMM ER [95% CI] = 64.8% [56.6, 72.2], Tukey’s p = 0.0005) and *An*. *gambiae s*.*s*. (EMM ER = 83.7% [59.0, 94.9], Tukey’s p = 0.012) were significantly endophagic. During this season, ER of other species were not different than 50% (Tukey’s p>0.25).

During dry hot season, EMM ERs of *An*. *funestus s*.*s* and *An*. *gambiae s*.*s* decreased to 56.2% [35.5, 75.0] and 71.1% [48.3, 86.6], respectively, no longer different than 50% (Tukey’s p = 0.56 and Tukey’s p = 0.069, respectively) but the numbers of individuals collected were small (n = 24 and n = 22, respectively) (Table 2). During this season, ER of other species were also not different than 50% (Tukey’s p>0.14).

Compared to dry hot season, the ER of *An*. *coluzzii* increased significantly (RR = 0.61 [0.41, 0.89], Tukey’s p = 0.0066) during rainy season to 59.3% [54.7, 63.8] that was significantly higher than 50% (Tukey’s p = 0.0001). During this season, ER of other species were not different than 50% (Tukey’s p > 0.12).

**Fig 3:**
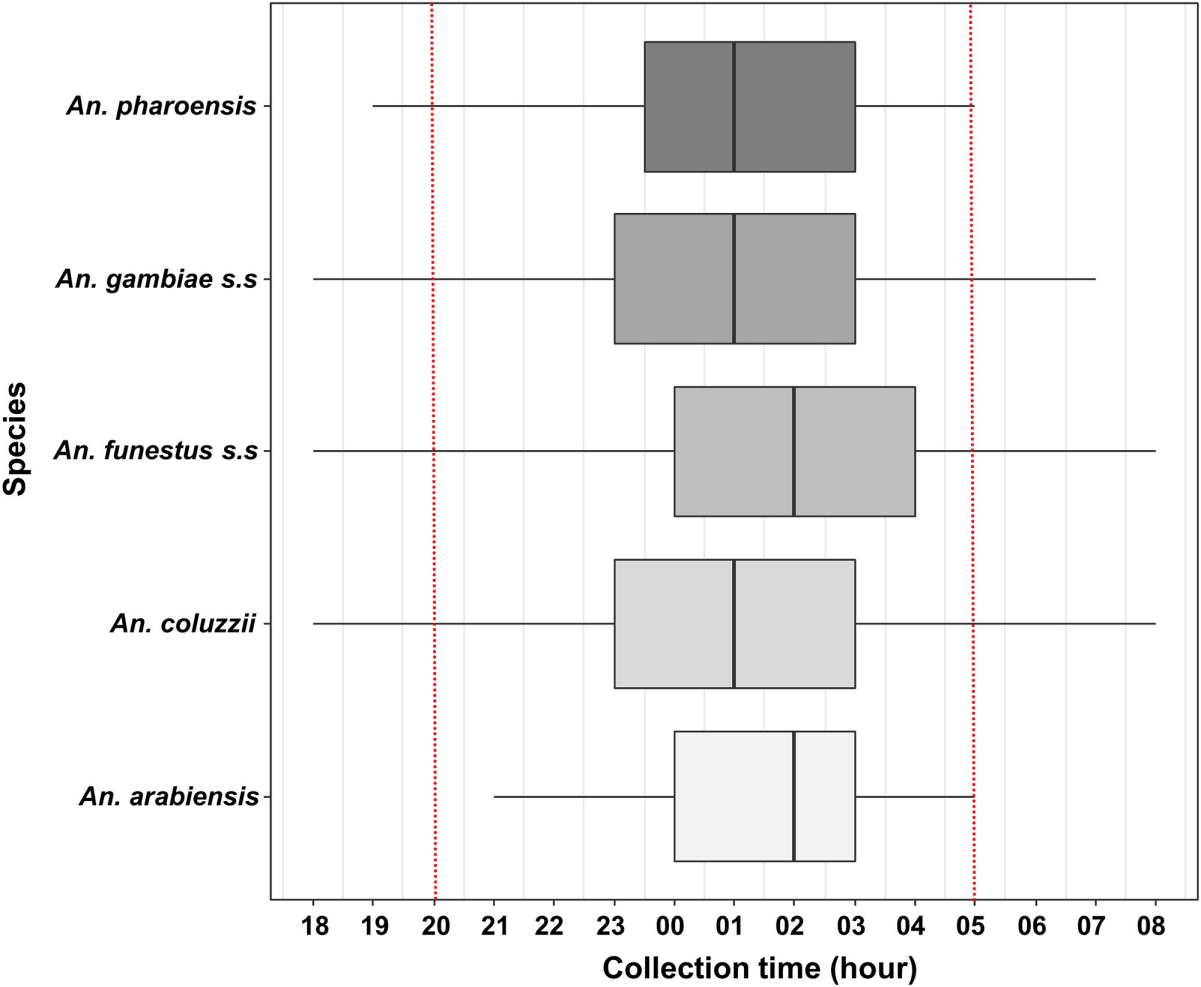
Median catching time of *Anopheles*. Boxes indicate 1st-3rd quartile and median hours of biting activity. Whiskers indicate 2.5–97.5 percentiles. Red dashed line indicated time at which 50% of the population are asleep (20h) and waked up (05h).

The median catching times of *An*. *coluzzii*, *An*. *gambiae s*.*s*, and *An*. *pharoensis* were recorded between 01:00 and 02:00 while those of *An*. *funestus s*.*s* and *An*. *arabiensis* were recorded one hour later (between 02:00 and 03:00; Fig 3). These differences were significant between *An*. *funestus s*.*s* and both *An*. *coluzzii* and *An*. *gambiae s*.*s* (Dunn’s multiple comparison test p-values = 0.01 and 0.004, respectively). *An*. *coluzzii*, *An*. *gambiae s*.*s*, *An*. *pharoensis* and *An*. *funestus s*.*s* showed earlier biting activity (beginning at 18:00) than *An*. *arabiensis* (beginning at 21:00). A late biting activity (after 06:00) was observed with *An*. *coluzzii*, *An*. *gambiae s*.*s* and *An*. *funestus s*.*s* (Fig 3).

### *Plasmodium* infection and transmission intensity

We analyzed a total of 1808 head-thoraxes of *Anopheles* for the research of *P*. *falciparum* infection. The overall sporozoite infection rate (SIR) was 0.07 [0.02–0.14] (19/272), 0.06 [0.01–0.11] (19/275) and 0.06 [0.00–0.70] (81/1261) in dry cold season, dry hot season and rainy season, respectively (Table 3). No *An*. *nili*, *An*. *pharoensis*, *An*. *rufipes* or *An*. *squamosus* individuals tested were infected. The SIR did not vary significantly among seasons (Likelihood ratio test χ^2^ = 4.47, df = 2, p = 0.1). The SIR of *An*. *gambiae s*.*s* was lower than that of *An*. *funestus s*.*s* (OR = 0.20 [0.05, 0.90], Tukey’s p = 0.03). No other differences between species have been evidenced (Tukey’s p-values > 0.23). The overall SIR both indoors and outdoors were included in (S1 Table). We collected infectious *Anopheles spp*. mosquitoes in seven villages out of the 27 surveyed (S1–S3 Figs).

**Table 3:** Entomological transmission parameters.

Species	Dry cold season		Dry hot season		Rainy season	
	SIR (%)	EIR	SIR (%)	EIR	SIR (%)	EIR
*An*. *arabiensis*	0.00	0.000	12.50	0.005	1.61	0.005
*An*. *coluzzii*	14.29	0.009	7.73	0.079	6.35	0.264
*An*. *gambiae s*.*s*	11.76	0.009	4.55	0.005	8.04	0.106
*An*. *funestus s*.*s*	6.79	0.069	0.00	0.000	0.00	0.000
*An*. *nili*	0.00	0.000	-	-	0.00	0.000
*An*. *pharoensis*	0.00	0.000	0.00	0.000	0.00	0.000
*An*. *rufipes*	0.00	0.000	-	-	-	-
*An*. *squamosus*	-	-	-	-	0.00	0.000
**Mean**	**0.07**	**0.087**	**0.06**	**0.089**	**0.06**	**0.375**
**[95% CI]**	[0.02–0.14]	[0.06–0.10]	[0.01–0.11]	[0.05–0.11]	[0.00–0.7]	[0.30–0.44]

SIR: Sporozoite infection rate; EIR: entomological inoculation rate; [95% CI]: 95% Confidence interval.

Entomological inoculation rate (EIR) was 0.375 [0.30–0.44] infected bites per human per night (ib.h^-1^.n^-1^) during the rainy season significantly higher than 0.087 ib.h^-1^.n^-1^ [0.06–0.10] measured during the dry cold season (RR = 0.25 [0.11, 0.56], Tukey’s p = 0.0002) and 0.089 ib.h^-1^.n^-1^ [0.05–0.11] measured during the dry hot season (RR = 0.23 [0.10, 0.51], Tukey’s p = 0.0001) (Table 3).

### Physiological age

We dissected 966 *Anopheles* for determination of parous rate. *Anopheles* parous rate was 76.00% [68.51–83.48], 78.80% [72.28–85.32] and 66.66% [63.14–70.18] in the dry cold season, dry hot season and rainy season respectively (S2 Table). These values of parous rate were used to calculated the percentage of *Anopheles* which could be found beyond the epidemiologically dangerous age and could therefore transmit malaria parasites. The percentages of *Anopheles* beyond the epidemiologically dangerous age were 22.10%, 27.16% and 10.78% in the dry cold, dry hot and rainy seasons, respectively. The average parous rate of *Anopheles* mosquitoes during both cold and hot dry seasons was significantly higher than during the rainy season (OR = 1.8 [1.008–3.21], Tukey’s p = 0.046 and OR = 1.95 [1.134–3.36], Tukey’s p = 0.011, respectively). Overall, parous rate was 76.38%, 68.97%, 67.24%, 72.65%, 77.77% and 100% for *An*. *funestus s*.*s*, *An*. *arabiensis*, *An*. *coluzzii*, *An*. *gambiae s*.*s*, *An*. *pharoensis* and *An*. *nili* respectively (S2 Table). The parous rate did not differ significantly among the species (Likelihood ratio test χ ^2^ = 2.51, df = 3, p = 0.47).

### Frequencies of L1014F *kdr*, L1014S *kdr* and G119S *ace-1* mutations in *An*. *gambiae* s.l.

Numbers of individuals of each genotype of the three mutations and their frequencies in *An*. *arabiensis*, *An*. *gambiae s*.*s*. and *An*. *coluzzii* are presented for each season (Table 4). We were not able to find a significant relationship between infectiousness and the genotypes of the *kdr-w* mutation (χ^2^ = 1.44, df = 2, p = 0.48), *kdr-e* mutation (Fisher’s exact test, p = 0.16) and *ace-1* mutation (Fisher’s exact test, p = 0.17) in the *An*. *gambiae s*.*l*. population.

**Table 4:** Allele frequency of *kdr* L1014F, *kdr* L1014S and *ace-1* G119S mutations in *Anopheles gambiae* s.l. populations.

			Genotypes *kdr-w*						Genotypes *kdr-e*						Genotypes *ace-1*				
Species	Period	N	SS	RS	RR	f (1014F)	p(HW)	N	SS	RS	RR	f(1014S)	p(HW)	N	SS	RS	RR	f(119S)	p(HW)
***An*. *arabiensis***	**Dry cold season**	4	0	4	0	0.500^a^	0.3141	4	4	0	0	0.000^a^	-	3	3	0	0	0.000^a^	-
	**Dry hot season**	8	5	3	0	0.188^a^	1.0000	8	8	0	0	0.000^a^	-	8	6	2	0	0.125^b^	1.0000
	**Rainy season**	56	35	18	3	0.214^a^	0.6978	53	22	31	0	0.292^b^	0.0022	61	61	0	0	0.000^a^	-
***An*. *coluzzii***	**Dry cold season**	14	1	9	4	0.607^ab^	0.3164	14	14	0	0	0.000^a^	-	14	12	2	0	0.071^a^	1.0000
	**Dry hot season**	219	37	63	119	0.687^a^	0.0000	218	217	1	0	0.002^a^	-	219	211	8	0	0.018^a^	1.0000
	**Rainy season**	823	176	389	258	0.550^b^	0.1938	743	453	289	1	0.196^b^	0.0000	888	849	39	0	0.022^a^	1.0000
***An*. *gambiae s*.*s***	**Dry cold season**	17	0	1	16	0.971^a^	-	17	17	0	0	0.000^a^	-	16	9	7	0	0.219^a^	0.5446
	**Dry hot season**	22	8	2	12	0.591^b^	0.0000	22	22	0	0	0.000^a^	-	22	17	5	0	0.114^a^	1.0000
	**Rainy season**	258	16	26	216	0.888^a^	0.0000	226	213	13	0	0.031^a^	1.0000	282	179	97	6	0.193^a^	0.1253

N: number of mosquitoes; SS: homozygous susceptible; RS: heterozygous; RR: homozygous resistant; *kdr-w*: kdr-west; *kdr-e*: kdr- east; f(1014F): frequency of the 1014F resistant *kdr* allele; f(1014S): frequency of the 1014S resistant *kdr* allele; f(119S): frequency of the 119S resistant *ace-1* allele; p(HW): exact Hardy-Weinberg test p-value; ‘-’: not determined; In each species and mutation, allelic frequencies carrying the same superscript letter do not differ significantly (G-test p > 0.05).

In *An*. *arabiensis*, the *kdr-w* mutation did not vary significantly among seasons (exact G test p-values > 0.15) nor among villages (exact G test p-values > 0.08). The population did not differ significantly from the Hardy-Weinberg equilibrium (HWE) whatever the season (exact HW test p > 0.31).

The *kdr-w* frequency in *An*. *coluzzii* during the dry cold season was 0.61, it increased (not significantly, exact G test p = 0.41) to 0.69 during dry hot season and then decreased significantly to 0.55 during the rainy season (exact G test p<0.001). During the dry hot season, when the frequency of the *kdr-w* mutation was the highest, the population was not at the HWE (exact HW test p< 0.001) due to heterozygote deficiency (exact HW test p<0.001) observed in the village of Diagnon where most of the individuals (131/220) where collected.

In *An*. *gambiae s*.*s*, the *kdr-w* mutation frequency was 0.97 during dry cold season and significantly decrease to 0.59 in dry hot season (exact G test p < 0.001). During the rainy season when mosquito densities were very high, the *kdr-w* frequency rose up to 0.88. That was significantly higher than during dry hot season (exact G test p < 0.001) but not different than during dry cold season (exact G test p = 0.12). This population was not at the HWE for the *kdr-w* mutation (exact HW test p < 0.001) due to heterozygote deficiency (exact HW test p < 0.001). Heterozygote deficiency was observed in most of the villages during each season (S2 File).

In *An*. *arabiensis*, the *kdr-e* mutation was detected only during the rainy season at a frequency of 0.30. The frequency of the *kdr-e* mutation did not vary significantly among villages (exact G test p > 0.10). A significant deviation to HWE was observed (exact HW test p = 0.002) due to heterozygote excess in rainy season (exact HW test p = 0.001). However, we did not observe this heterozygote excess at the village scale (S3 File).

In *An*. *coluzzii*, the *kdr-e* mutation was not detected during the dry-cold season and only one heterozygous individual was collected during the dry hot season corresponding to a frequency of 0.002. The frequency increased significantly to 0.12 during the rainy season (exact G test p < 0.001). A significant deviation to HWE was observed for the *kdr-e* mutation (exact HW test p < 0.001) due to heterozygote excess in most of the villages during each season (S4 File).

In *An*. *gambiae s*.*s*, the *kdr-e* mutation was detected only during the rainy season at a frequency of 0.03. The *kdr-e* mutation did not vary significantly among villages (exact G test p > 0.11). The population did not differ significantly from the HWE (exact HW test p > 0.05) in rainy season.

In *An*. *arabiensis*, the *ace-1* mutation was detected only during the dry hot season at a frequency of 0.12. The frequency of the *ace-1* mutation did not vary significantly among villages (exact G test p > 0.48). The population did not differ significantly from the HWE (exact HW test p > 0.05) in dry hot season.

The frequency of the *ace-1* allele in *An*. *coluzzii* was 0.07, 0.02 and 0.02 in dry cold, dry hot and rainy seasons, respectively. There were no significant difference in the *ace-1* allele frequency among seasons (exact G test p > 0.11) nor among villages (exact G test p > 0.13). The *ace-1* allele frequency in *An*. *coluzzii* did not differ from the HWE (exact HW test p > 0.05).

In *An*. *gambiae s*.*s*, the frequencies of the *ace-1* allele were 0.22, 0.11 and 0.19 in dry cold, dry hot and rainy seasons, respectively. The frequency did not vary significantly among seasons (exact G test p > 0.23) nor among villages (exact G test p > 0.07). The population did not differ significantly from the HWE (exact HW test p > 0.12) whatever the season.

Three hundred and fifty four (354) individuals *An*. *gambiae s*.*l* carried multiple insecticide resistance mechanisms. Two hundred and seven (207) carried at least one mutated allele for both *kdr-w* and *kdr-e* mutations (belonging the *An*. *gambiae s*.*s*, *An*. *coluzzii* and *An*. *arabiensi*s species). One hundred and forty-two (142) carried at least one mutated allele for both *kdr-w* and *ace-1* mutations (belonging the *An*. *gambiae s*.*s*, *An*. *coluzzii* and *An*. *arabiensi*s species) and 16 carried at least one mutated allele for both *kdr-e* and *ace-1* mutations (belonging the *An*. *gambiae s*.*s* and *An*. *coluzzii* species). Eleven (11) individuals carried the three mutations (belonging the *An*. *gambiae s*.*s* and *An*. *coluzzii* species).

## Discussion

This study showed that the malaria vector species and abundance in the Diébougou area varied significantly according to the season. *Anopheles funestus s*.*s* was the predominant vector during the dry cold season (January 2017) but most individuals were collected in one village (Diagnon) that is close to swamps on the edge of the dam of Bapla. *Anopheles funestus s*.*s* densities were ten times lower two month later during the dry hot season (March 2017) and it almost disappeared in June 2017 during the rainy season. *Anopheles funestus s*.*s* is known to breed in large permanent or semi-permanent pools preferentially with emergent vegetation on its margins [39, 40]. In Burkina Faso, two chromosomal forms of *An*. *funestus* have been described named Folonzo and Kiribina [41, 42]. Folonzo form of *An*. *funestus* is mainly associated with the presence of water reservoirs containing natural vegetation, such as swamps. The swamps near Diagnon are upstream from the Bapla dam and become dry at the end of the dry season. Provided that *An*. *funestus* in our study area is of the Folonzo form, this may explain why *An*. *funestus s*.*s* almost disappeared during the dry hot season and until swamps become green and flooded again.

*Anopheles coluzzii* was shown at very low densities during the dry cold season but became the predominant malaria vector species from the dry hot season (March 2017) to the rainy season (June 2017). During the dry hot season, most of individuals were collected in Diagnon, the same village where *An*. *funestus s*.*s* densities were simultaneously falling. In Burkina Faso, *An*. *coluzzii* is known to breed in permanent or semi-permanent breeding sites [43, 44]. Its presence in Diagnon during the dry season is certainly linked to the dam. However, it is not clear why *An*. *coluzzii* was present in very low densities during January 2017 and became numerous two months later. We hypothesize that the reduction of the breeding sites favorable to *An*. *funestus s*.*s* may have increased *An*. *coluzzii* competitiveness against *An*. *funestus s*.*s* around the Bapla dam. This result is in accordance with the high variability of *An*. *coluzzii* densities during the dry season as described by Dao *et al*. [45] in the neighboring Mali.

Densities of *An*. *gambiae s*.*s*. and *An*. *arabiensis* were low during both dry (cold and hot) seasons and increased substantially during the rainy season (in the largest extent for *An*. *gambiae s*.*s*.). This is consistent with the preference of both these species to breed in temporary rain-dependent pools and puddles [40, 43, 46].

These four species (*An*. *funestus s*.*s*, *An*. *coluzzii*, *An*. *gambiae s*.*s* and *An*. *arabiensis)* were responsible for all the *P*. *falciparum* transmission recorded in this study. Sporozoite infection rate (SIR) did not vary among seasons but EIR increase from an average of about 3 infected bites per human per month in dry (cold and hot) seasons to more than 10 bites per human per month during the rainy season. The values of EIR recorded in our study are consistent with previous works in the same region of Burkina Faso [47–49]. Such levels of transmission (EIR > 100 infected bites per person per year) are relatively high when put in the African context [50]. As SIR was stable over the study, the increase in EIR in rainy season was mathematically due to the increase in vectors densities. According to our results, malaria transmission (as measured by EIR) persists year round in the study area despite high spatial disparities. Indeed, during the dry cold and dry hot seasons, the spatial distribution of *An*. *funestus s*.*s*. and *An*. *coluzzii*, that were the main malaria vectors, was restrained to a small number of villages that concentrated malaria transmission. During the rainy season, the spatial distribution of malaria vector (with predominance of *An*. *coluzzii* and *An*. *gambiae s*.*s*.) was more homogeneous.

In addition to these well-known primary malaria vectors, we recorded the presence of four other *Anopheles* species in low densities (*An*. *nili*, *An*. *pharoensis*, *An*. *rufipes*, *An*. *squamosus*). While we were not able to detect *Plasmodium* parasites in the few individuals belonging to these species, they are all potential vectors of *Plasmodium* [40, 51–55]. Because they are considered secondary vectors [56, 57] (due to their main trophic behavior including exophagy and/or zoophagy) or found in low densities, these species are often neglected in most of the recent studies carried out in Burkina Faso [58]. However, these vectors could possibly maintain residual levels of transmission [57, 59]. Indeed, drastic reduction of the major vector densities may free up their ecological niches for secondary vectors [60–63]. Therefore, secondary vectors deserve particular attention [64]. Their bionomics and competence for *P*. *falciparum* transmission remain to be investigated.

Regarding the behavior of the main malaria vector species, we observed that *An*. *coluzzii*, *An*. *gambiae s*.*s* and *An*. *funestus s*.*s* were slightly but mainly endophagic (50 to 65% depending on the season) that is in accordance with previous results recorded in another location in southwest Burkina Faso [47]. This indicates that indoor vector control measures (such as LLINs and IRS) are expected to target a significant part of the vector population but probably insufficient to stop the transmission. The peak of aggressiveness of the main *Anopheles* species occurred during the second part of the night. This observation is consistent with the usual patterns of hourly biting aggressiveness of these species [65]. This period corresponds to the moments of deep sleep of the human populations who are potentially protected by LLINs [46, 66]. However, we observed early- and late-biting phenotypes in the main malaria vector species (*An*. *coluzzii*, *An*. *funestus s*.*s*. and *An*. *gambiae s*.*s*). These phenotypes might mediate residual transmission and we might expect to see them selected by the massive use of insecticide-based vector control tools such as LLINs [67, 68]. It is therefore crucial to monitor malaria vector behavior in this area when implementing malaria vector control strategies in order to track the possible emergence of behavioral resistances.

This study showed high parous rates of malaria vectors (> 60%) in the study area, regardless of the season. The parous rate was significantly lower in the rainy season than in the cold and hot dry seasons, indicating that young females were more prevalent in the rainy season. The lower parous rate of *Anopheles* vectors during the rainy season may be due to the presence of more breeding habitats which yield more nulliparous mosquitoes in vector populations [69]. It is also possible that a higher usage of LLINs during the rainy season as a result of a higher nuisance and lower temperatures might result in smaller proportions of parous vectors [70]. The three target-site mutations *kdr-w*, *kdr-e* and *ace-1* that were tested for were detected in the three species of the *An*. *gambiae* complex but at varying frequencies among species and seasons. In June 2017 during the rainy season (when the populations were most abundant), the *kdr-w* mutation frequency was very high in *An*. *gambiae s*.*s*. (almost fixed, f = 0.88). Since the discovery of this mutation [26] in *An*. *gambiae s*.*s*, the *kdr-w* frequencies have always been high in this species in Southwest Burkina Faso [8, 10, 71] indicating that the mutation has a low cost for this species or that strong selection pressures occur. Selection pressure likely to occur might be due to vector control (universal distribution of LLINs is implemented in Burkina Faso since 2010 [72]) and massive use of pesticides in agriculture [73]. Indeed, the intensive use of insecticides in cotton cultivation might be a major factor driving the selection of pyrethroid-resistant specimens in Southwest Burkina Faso [10]. Most of the populations of *An*. *gambiae s*.*s*. from this study showed heterozygote deficiency for the *kdr-w* mutation that could indicate a Wahlund effect resulting from the presence of several populations within each given village. This might be explained by the colonization of temporary breeding sites by different vector populations during the rainy season as evidence the annual reappearance of *An*. *gambiae s*.*s*. in the Sahel after the dry season [45]. To note that no disequilibrium was observed for the *kdr-e* and *ace-1* mutations in this species indicating that these populations, if they exists, resemble the local population in terms of genotypic composition for these loci.

During the rainy season, the *kdr-w* mutation frequency was lower in *An*. *coluzzii* (f = 0.55) and *An*. *arabiensis* (f = 0.21) than in *An*. *gambiae s*.*s*. But in counterpart, both *An*. *coluzzii* (f = 0.19) and *An*. *arabiensis* (f = 0.29) showed higher prevalence of the *kdr-e* mutations than *An*. *gambiae s*.*s*. (f = 0.03). In *An*. *coluzzii* and *An*. *arabiensis*, a heterozygote excess was observed for the *kdr-e* mutation. This disequilibrium may result from a heterozygote advantage as it has been observed in mating in *An*. *coluzzii* specimen from Southwest Burkina Faso carrying the *kdr-w* mutation [74]. The causes of this disequilibrium remain to be explored.

Both *kdr* mutations are well “implanted” in the vector population of our study area. These mutations provide physiological resistance to the pyrethroids insecticides, the only family of compounds authorized to impregnate LLINs. Although the impact of pyrethroids resistance on the efficacy of malaria control remains disputable [75], the development and the implementation of resistance management strategies need to be encouraged.

Regarding the *ace-1* mutation that confers resistance to carbamates and organophosphates insecticides, it was present in the three species at a moderate frequency in *An*. *gambiae s*.*s*. but at very low frequencies in *An*. *coluzzii* and *An*. *arabiensis*. In Burkina Faso, the three species may express high frequencies of the *ace-1* mutation depending on the location [76]. In our study area, *An*. *gambiae s*.*s*. seems to suffer more selection pressure possibly due to a wide use of carbamates and organophosphates particularly for cotton growing.

Recent studies in Tanzania and Senegal showed a relationship between the genotype for the *kdr* mutation of wild *Anopheles gambiae s*.*l*. and *Plasmodium falciparum* infection [77]. The mosquitoes carrying the mutation were more infected than those carrying the wild alleles. In contrast, we were not able to show such a relationship whatever the mutation studied (*kdr-w*, *kdr-e* or *ace-1*). This may be explained by the complex interactions occurring between insecticide resistance and *Plasmodium* infection as summarized in [78]. Indeed, while an insecticide resistance mutation may be associated with increased vector competence [79], the infection by *Plasmodium falciparum* tends to decrease survival of vectors carrying this mutation [80].

## Conclusions

Malaria transmission in the Diébougou area was mainly due to *An*. *funestus s*.*s*, *An*. *coluzzii*, *An*. *gambiae s*.*s* and *An*. *arabiensis* with high spatio-temporal heterogeneities. The entomological indicators of malaria transmission were high despite the presence of LLINs. We observed early and late biting phenotypes in the main malaria vector species. These phenotypes might mediate residual transmission. Three mutations *kdr-w*, *kdr-e* and *ace-1* were present in the three species of the *An*. *gambiae* complex with high frequency variability between species. These data will be used to evaluate the impact of complementary tools to LLINs in an upcoming RCT.

## Supporting information

S1 Fig Map of infectious *Anopheles spp* collected par village during dry cold season. Top-left box shows the location of the Diébougou health district in Burkina Faso. Background was obtained freely from openstreetmap.org.
(TIF)

S2 Fig Map of infectious *Anopheles spp* collected par village during dry hot season. Top-left box shows the location of the Diébougou health district in Burkina Faso. Background was obtained freely from openstreetmap.org.
(TIF)

S3 Fig Map of infectious *Anopheles spp* collected par village during rainy season. Top-left box shows the location of the Diébougou health district in Burkina Faso. Background was obtained freely from openstreetmap.org.
(TIF)

S1 Table Mean estimates of the Sporozoite Infection Rate (SIR) according to locations (indoors and outdoors). (DOCX)

S2 Table Parous rate of *Anopheles*. (DOCX)

S1 File Results of the final models. (PDF)

S2 File Hardy Weinberg test when H1 = heterozygote deficit in *An*. *gambiae s*.*s* populations. (TXT)

S3 File Hardy Weinberg test when H1 = heterozygote excess in *An*. *arabiensis* populations. (TXT)

S4 File Hardy Weinberg test when H1 = heterozygote deficit in *An*. *coluzzii* populations. (TXT)
