# Supplementary figures and images for: Anopheles bionomics, insecticide resistance and malaria transmission in southwest Burkina Faso: A pre-intervention study
Source: PLoS One. 2020 Aug 3;15(8):e0236920. doi: 10.1371/journal.pone.0236920 (PMC7398507; doi:10.1371/journal.pone.0236920)

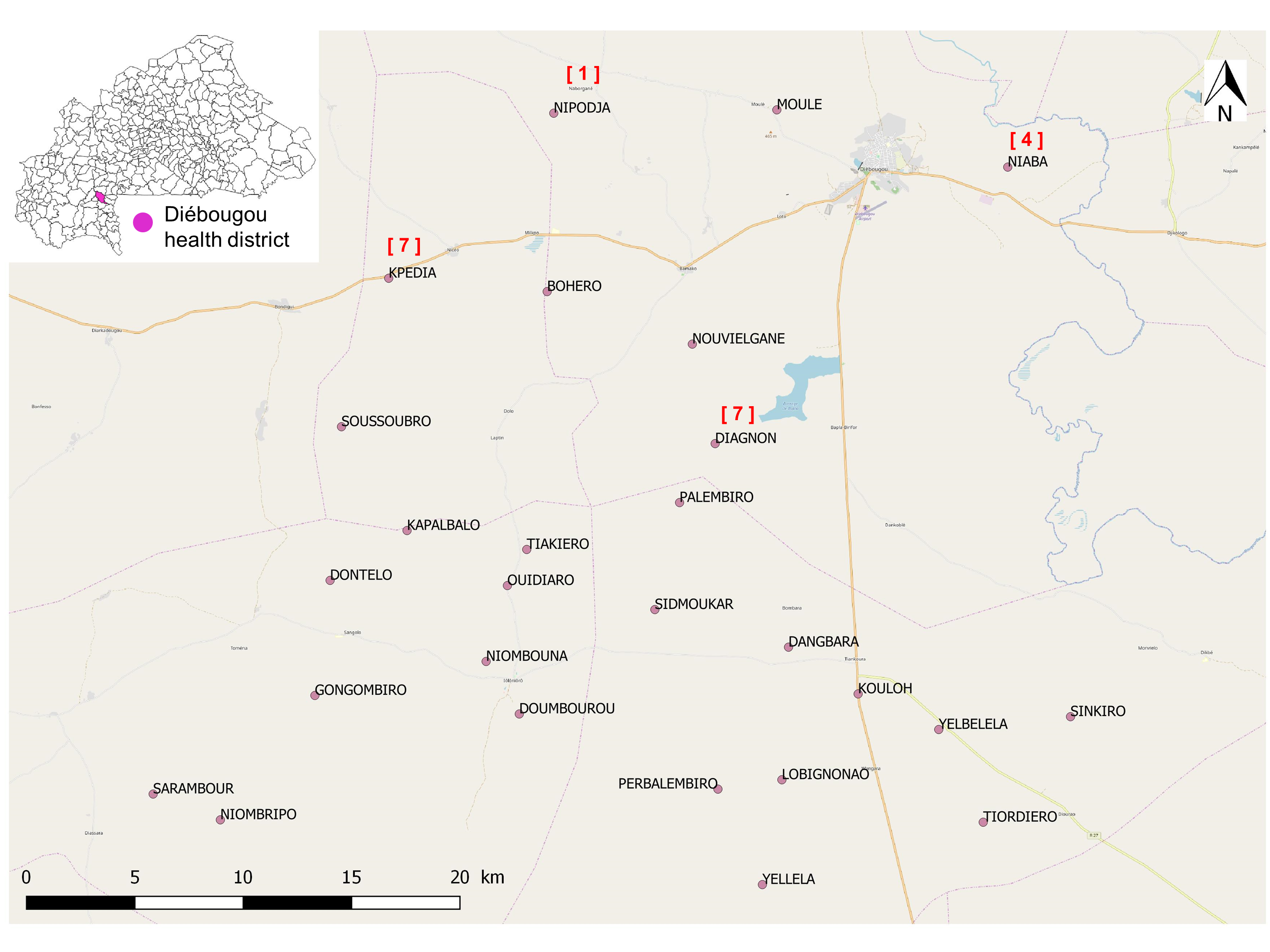

Supplement: S1 Fig — Top-left box shows the location of the Diébougou health district in Burkina Faso. Background was obtained freely from openstreetmap.org. (TIF) [file pone.0236920.s001.tif]

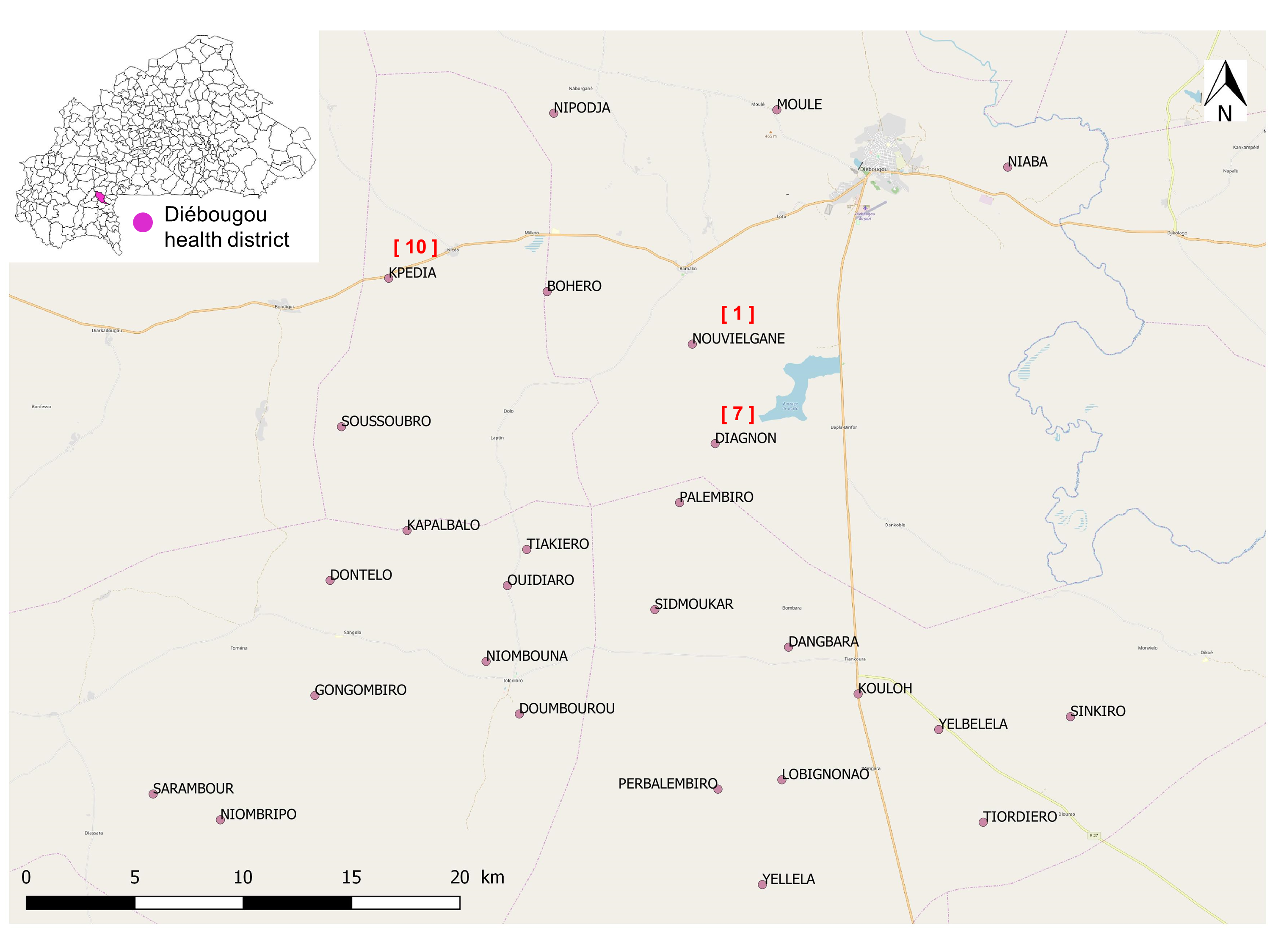

Supplement: S2 Fig — Top-left box shows the location of the Diébougou health district in Burkina Faso. Background was obtained freely from openstreetmap.org. (TIF) [file pone.0236920.s002.tif]

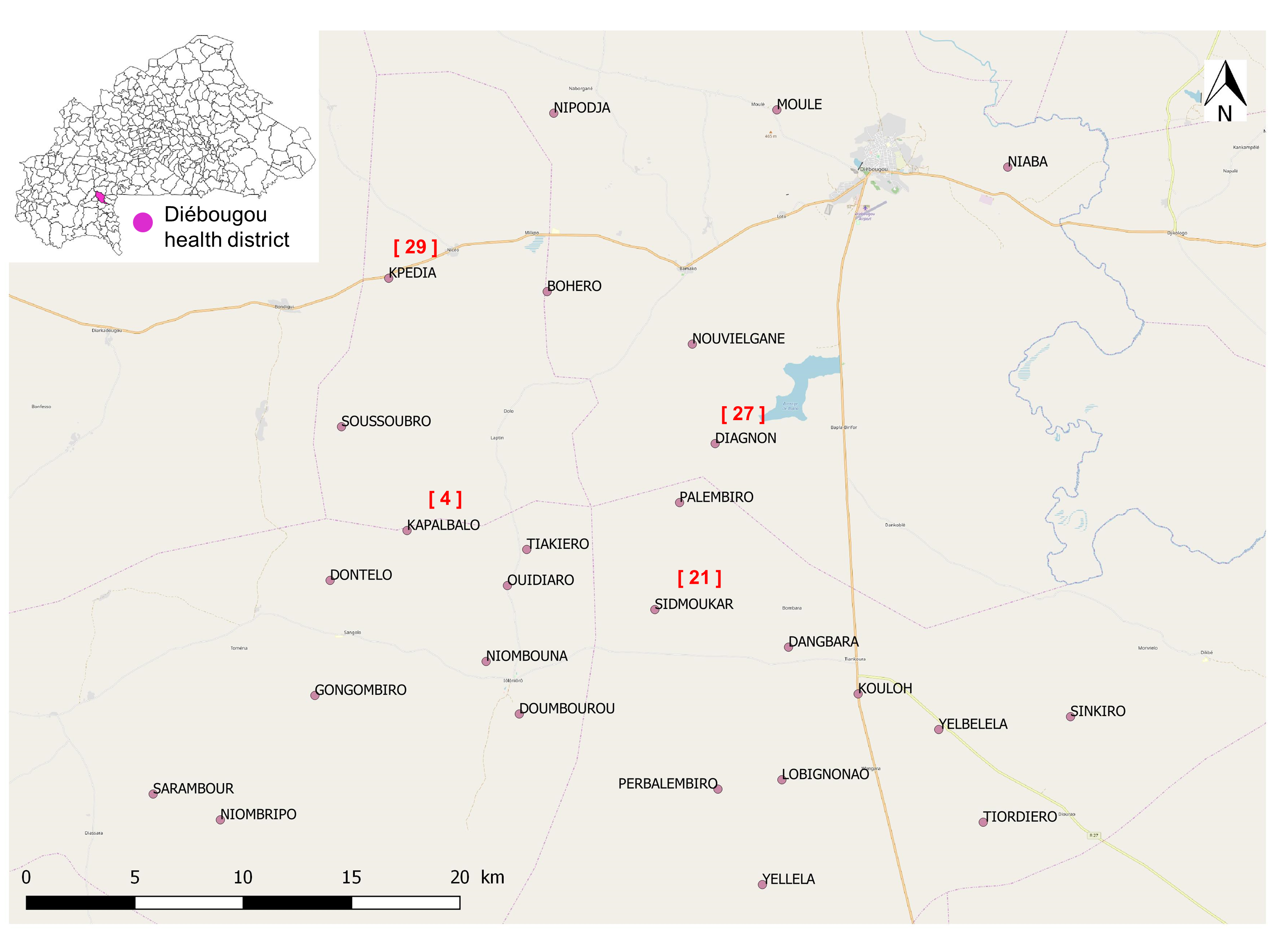

Supplement: S3 Fig — Top-left box shows the location of the Diébougou health district in Burkina Faso. Background was obtained freely from openstreetmap.org. (TIF) [file pone.0236920.s003.tif]
