# Supplementary material for: Anopheles bionomics, insecticide resistance and malaria transmission in southwest Burkina Faso: A pre-intervention study
Source: PLoS One. 2020 Aug 3;15(8):e0236920. doi: 10.1371/journal.pone.0236920 (PMC7398507; doi:10.1371/journal.pone.0236920)
Supplement: S1 Table — (DOCX) [file pone.0236920.s004.docx]

**S1 Table. Mean estimates of the sporozoite infection rate (SIR) according to locations (indoors and outdoors)**

| **Species** | **Location** | **Dry cold season** | | |  | **Dry hot season** | | |  | **Rainy season** | | |
| --- | --- | --- | --- | --- | --- | --- | --- | --- | --- | --- | --- | --- |
|  |  |  | | |  |  | | |  |  | | |
|  |  | No. Positive | No. Tested | Mean SIR [95% CI] |  | No. Positive | No. Tested | Mean SIR [95% CI] |  | No. Positive | No. Tested | Mean SIR [95% CI] |
| *An. arabiensis* | Indoor | 0 | 2 | 0.00 [0.00-0.65] |  | 0 | 2 | 0.00 [0.00-0.65] |  | 0 | 31 | 0.00 [0.00-0.11] |
| *An. coluzzii* |  | 1 | 5 | 0.20 [0.03-0.62] |  | 5 | 106 | 0.04 [0.02-0.10] |  | 39 | 525 | 0.07 [0.05-0.09] |
| *An. gambiae s.s* |  | 2 | 14 | 0.14 [0.04-0.39] |  | 1 | 16 | 0.06 [0.01-0.28] |  | 14 | 159 | 0.08 [0.05-0.14] |
| *An. funestus s.s* |  | 10 | 146 | 0.06 [0.03-0.12] |  | - | 14 | 0.00 [0.00-0.21] |  | 0 | 3 | 0.00 [0.00-0.56] |
| *An. nili* |  | 0 | 1 | 0.00 [0.00-0.79] |  | - | 0 | - |  | 0 | 1 | 0.00 [0.00-0.79] |
| *An. pharoensis* |  | 0 | 3 | 0.00 [0.00-0.56] |  | - | 0 | - |  | 0 | 2 | 0.00 [0.00-0.65] |
| *An. rufipes* |  | 0 | 1 | 0.00 [0.00-0.79] |  | - | 0 | - |  | - | 0 | - |
| *An. squamosus* |  | - | 0 | - |  | - | 0 | - |  | - | 0 | - |
| **Total** |  | **13** | **172** | **0.07 [0.04-0.12]** |  | **6** | **138** | **0.04 [0.02-0.09]** |  | **53** | **756** | **0.07 [0.05-0.09]** |
|  |  |  |  |  |  |  |  |  |  |  |  |  |
| *An. arabiensis* | Outdoor | 0 | 2 | 0.00 [0.00-0.65] |  | 1 | 6 | 0.16 [0.03-0.56] |  | 1 | 31 | 0.03 [0.00-0.16] |
| *An. coluzzii* |  | 1 | 9 | 0.11 [0.01-0.43] |  | 12 | 114 | 0.10 [0.06-0.17] |  | 18 | 372 | 0.04 [0.03-0.07] |
| *An. gambiae s.s* |  | 0 | 3 | 0.00 [0.00-0.56] |  | 0 | 6 | 0.00 [0.00-0.39] |  | 9 | 127 | 0.07 [0.03-0.12] |
| *An. funestus s.s* |  | 5 | 75 | 0.06 [0.02-0.14] |  | 0 | 10 | 0.00 [0.00-0.27] |  | 0 | 2 | 0.00 [0.00-0.65] |
| *An. nili* |  | - | 0 | - |  | - | 0 | - |  | 0 | 1 | 0.00 [0.00-0.79] |
| *An. pharoensis* |  | 0 | 11 | 0.00 [0.00-0.25] |  | 0 | 1 | 0.00 [0.00-0.79] |  | 0 | 6 | 0.00 [0.00-0.39] |
| *An. rufipes* |  | - | 0 | - |  | - | 0 | - |  | - | 0 | - |
| *An. squamosus* |  | - | 0 | - |  | - | 0 | - |  | 0 | 1 | 0.00 [0.00-0.79] |
| **Total** |  | **6** | **100** | **0.06 [0.02-0.12]** |  | **13** | **137** | **0.09 [0.05-0.15]** |  | **28** | **567** | **0.04 [0.03-0.07]** |

No.: number of mosquitoes; SIR: Sporozoite infection rate; [95% CI]: 95% Wilson’s confidence interval.
