# Supplementary material for: Anopheles bionomics, insecticide resistance and malaria transmission in southwest Burkina Faso: A pre-intervention study
Source: PLoS One. 2020 Aug 3;15(8):e0236920. doi: 10.1371/journal.pone.0236920 (PMC7398507; doi:10.1371/journal.pone.0236920)
Supplement: S1 File — (PDF) [file pone.0236920.s006.pdf]

S1_file: Final models

These are final models (after stepwise backward deletion) of HBR, ER, SR and EIR from the article entitled: Anopheles bionomics, insecticide resistance and malaria transmission in southwest Burkina Faso: a pre-intervention study by Dieudonne Diloma Soma, Barnabas Mahugnon Zogo, Anthony Some, Bertin NCho Tchiekoi, Domonbabele Francois de Sales Hien, Hermann Sie Pooda1, Sanata Coulibaly, Jacques Edounou Gnambani, Ali Ouari, Karine Mouline, Amal Dahounto, Georges Anicet Ouedraogo, Florence Fournet, Alphonsine Amanan Koffi, Cedric Pennetier, Nicolas Moiroux and Roch Kounbobr Dabire

Table below describes each parameter (variables) used in the models:

Variable type	Variable name	Data type	Variable description	Used in models
response	tot_ano	integer	cumulated nightly number of anopheles (all species) collected at each collection point	HBR
response	poste	binary	collection position (indoors or outdoors) of each individual anopheles	ER
response	pcrpf	binary	qpcr confirmed detection of P. falciparum DNA in individual anopheles	SIR
response	TauxParturite	binary	parity status of each individual anopheles	PR
response	sum	integer	cumulated nightly number of P. falciparum infected anopheles (all species) collected at each collection point	EIR
explanatory	enquete	factor	season with 3 modalities (1= dry cold, 2=dry hot, 3=rainy)	HBR, ER, SIR, PR, EIR
explanatory	poste	factor	collection position with 2 modalities (int=indoors, ext=outdoors)	HBR, EIR
explanatory	espece	factor	species with 4 modalities (Anarabiensis, Ancoluzzii, Anfunestus_ss, Angambiae_ss)	ER, SIR, PR
random	village	factor	unique identifier of village of collection	HBR, ER, SIR, PR, EIR
random	pt_capt	factor	unique identifier of collection site (4 sites per village)	HBR, ER, SIR, PR, EIR

The following table provides results of likelihood ratio test that drove the variable selection process:

Model	Predictors (fixed effects) tested	Likelihood ratio Test (after stepwise deletion)			Kept in the final models
		Chi	Df	p-value	
HBR	Season	272.8632	2	< 2.2e-16	Y
	Collection position	7.3658	1	0.006648	Y
	Season*position interaction	0.9405	2	0.624835	N
ER	Season	7.6544	3	0.05372	Y
	Species	2.1174	2	0.34691	Y
	Season*species interaction	13.6532	6	0.03376	Y
SIR	Season	4.4709	2	0.10695	N
	Species	9.6946	3	0.02135	Y
	Season*species interaction	4.4386	6	0.61754	N
PR	Season	8.9798	2	0.01122	Y
	Species	2.5147	3	0.47264	N
	Season*species interaction	7.269	6	0.29668	N
EIR	Season	26.9899	2	1.378e-06	Y
	Collection position	2.1329	1	0.1442	N
	Season*position interaction	5.3376	2	0.06933	N

Please find below the summaries of the final models that have been used for HBR, ER, SIR, PR and EIR analyses:

HBR (Human Biting Rate) model:

	**tot Ano**			
Predictors	*Log-Mean*	*std. Error*	*CI*	*p*
(Intercept)	-0.40	0.27	-0.92 – 0.13	0.138
enquete [2]	-0.11	0.18	-0.46 – 0.23	0.513
enquete [3]	1.72	0.13	1.46 – 1.98	**<0.001**
poste [ext]	-0.25	0.09	-0.43 – -0.07	**0.006**
N_{pt_capt}	108			
$N_{village}$	27			
Observations	648			
Marginal R^2 / Conditional R^2	0.208 / 0.603			

ER (Endophagy Rate) model:

	**poste**			
Predictors	*Log-Odds*	*std. Error*	*CI*	*p*
(Intercept)	0.23	1.04	-1.81 – 2.26	0.828
espece [Ancoluzzii]	-0.88	1.18	-3.20 – 1.44	0.455
espece [Anfunestus_ss]	0.38	1.05	-1.67 – 2.44	0.714
espece [Angambiae_ss]	1.41	1.22	-0.98 – 3.81	0.246
enquete [2]	-1.45	1.33	-4.05 – 1.15	0.273
enquete [3]	-0.22	1.07	-2.31 – 1.88	0.839
espece [Ancoluzzii] * enquete [2]	1.99	1.45	-0.85 – 4.83	0.170
espece [Anfunestus_ss] * enquete [2]	1.09	1.40	-1.65 – 3.83	0.434
espece [Angambiae_ss] * enquete [2]	0.71	1.55	-2.32 – 3.75	0.646
espece [Ancoluzzii] * enquete [3]	1.25	1.21	-1.13 – 3.63	0.302
espece [Anfunestus_ss] * enquete [3]	0.06	1.42	-2.73 – 2.84	0.968
espece [Angambiae_ss] * enquete [3]	-1.21	1.25	-3.67 – 1.25	0.335
N_{pt_capt}	91			
$N_{village}$	26			
Observations	1780			
Marginal R^2 / Conditional R^2	0.023 / 0.069			

SIR (Sporozoite Infection Rate) model:

	**pcrpf**			
Predictors	*Log-Odds*	*std. Error*	*CI*	*p*

(Intercept)	-6.14	1.32	-8.73 – -3.56	**<0.001**
espece [Ancoluzzii]	0.41	0.77	-1.11 – 1.92	0.599
espece [Anfunestus_ss]	0.22	0.82	-1.38 – 1.82	0.784
espece [Angambiae_ss]	1.02	0.79	-0.53 – 2.57	0.198
N_{pt_capt}	91			
$N_{village}$	26			
Observations	1780			
Marginal R^2 / Conditional R^2	0.008 / 0.667			

PR (Parity Rate) Model:

	Taux Parturite			
Predictors	*Log-Odds*	*std. Error*	*CI*	*p*
(Intercept)	1.36	0.25	0.87 – 1.86	**<0.001**
enquete [2]	0.08	0.30	-0.51 – 0.68	0.789
enquete [3]	-0.59	0.25	-1.07 – -0.10	**0.017**
N_{pt_capt}	81			
Observations	956			
Marginal R^2 / Conditional R^2	0.023 / 0.065			

EIR (Entomological Inoculation Rate) model:

	sum			
Predictors	*Log-Mean*	*std. Error*	*CI*	*p*
(Intercept)	-9.05	1.97	-12.90 – -5.20	**<0.001**
enquete [2]	-0.08	0.38	-0.84 – 0.67	0.825
enquete [3]	1.34	0.32	0.70 – 1.97	**<0.001**
N_{pt_capt}	108			
$N_{village}$	27			
Observations	648			
Marginal R^2 / Conditional R^2	0.009 / 0.813			
